# A Standards‐Based, Cloud‐Hosted CDS System for Gonorrhea Treatment and HIV Screening

**DOI:** 10.1002/lrh2.70104

**Published:** 2026-07-30

**Authors:** Edna Shenvi, Aziz Boxwala, Sarah Shaw, Andre Berro, Saugat Karki, Matthew Pooser, Jane E. Yang, Gema Dumitru, Jeanne Ocampo, Amrita Tailor, Sanjat Kanjilal, Carlos Paredes, Ritche Hao, Nitu Kashyap, David Liebovitz, Alejandro Pérez

**Affiliations:** ^1^ Elimu Informatics El Cerrito California USA; ^2^ Public Health Informatics Institute Atlanta Georgia USA; ^3^ Centers for Disease Control and Prevention Atlanta Georgia USA; ^4^ Harvard Medical School Boston Massachusetts USA; ^5^ Yale New Haven Health New Haven Connecticut USA; ^6^ Emory Healthcare Atlanta Georgia USA; ^7^ Northwestern University Feinberg School of Medicine Chicago Illinois USA

**Keywords:** clinical decision support, external CDS, HIV, interoperability, public health, sexually transmitted infection

## Abstract

**Objective:**

*Neisseria gonorrhoeae* (or gonococcus, GC) infections have increased in incidence and antibiotic resistance, prompting updates to management guidelines. Additionally, patients with recent GC infections have been identified as a target population for human immunodeficiency virus (HIV) screening and prevention. With the rapidly changing nature of such biomedical knowledge, there is an imperative to adopt public health guidelines speedily to deliver knowledge to clinicians at the point of care. Therefore, the Public Health Informatics Institute and the United States Centers for Disease Control and Prevention sought to create a scalable clinical decision support (CDS) solution that could be easily integrated using interoperability standards with widely used electronic health record (EHR) systems in the United States.

**Methods:**

A project team including informaticians and clinical partners developed a cloud‐hosted CDS system, with rule logic written in the Health Level Seven (HL7) Clinical Quality Language and using the HL7 CDS Hooks protocol. The system was designed to present alerts to clinicians who, based on available data, appeared to be treating uncomplicated GC with doses or agents that were not recommended based on the patient's weight, allergies, and other characteristics. Actionable suggestions were offered for the correct regimen, and HIV testing, if applicable.

**Results:**

After extensive testing, the system was successfully integrated with the EHR system at two academic medical institutions. Subsequently, a three‐month pilot was conducted in emergency and urgent care settings.

**Conclusions:**

There were limitations related to functionality, workflow issues, and data quality. This successful implementation, however, demonstrates the feasibility of this approach for select use cases of CDS, and yields further understanding of the requirements for developing scalable solutions for disseminating public health guidance.

## 1 Introduction

*Neisseria gonorrhoeae* (or gonococcus, GC) infections have increased in incidence. In the United States (US), the per capita incidence increased by 32% from 2006 to 2019 [1]. At the same time, resistance to antibiotics used for the treatment of GC infections has increased [2]. This change in susceptibility to recommended treatments in the 2015 publication of guidelines for management of sexually transmitted infections (STI) [3] prompted their revision. In 2020 and 2021 updates to STI and gonorrhea management guidance [4, 5], the recommendation was changed to increase the dose of ceftriaxone for uncomplicated urogenital/anorectal or pharyngeal infections from 250 to 500 mg. Untreated or inadequately treated GC can have severe impacts on reproduction (e.g., pelvic inflammatory disease), facilitate human immunodeficiency virus (HIV) transmission, and increase the risk of antibiotic resistance of GC. Recent updates to HIV prevention guidelines [6] identify individuals with GC in the prior 6 months as having higher risk of contracting HIV, and therefore a target audience for HIV screening and prevention.

The Public Health Informatics Institute (PHII) and the United States Centers for Disease Control and Prevention (CDC) sought to develop and pilot a clinical decision support (CDS) solution as an electronic health record (EHR)‐agnostic, standards‐based application for GC treatment, HIV testing, and provision of pre‐exposure prophylaxis (PrEP) information. The project was built on 5 years of work to evaluate the feasibility and usability in real‐world clinical settings of CDS for the management of STI. In a prior effort, the GC treatment guidelines were successfully implemented as workflow‐integrated CDS tools [7]. A limitation of that implementation was that CDS was created using features proprietary to an EHR product. Therefore, these CDS tools could not be reused at sites with other EHR systems. Another concern with this approach is that when the guidelines are updated, the EHR‐native CDS tool must be updated at each site manually. Both issues inhibit the rapid and easy implementation of CDS.

However, there is an imperative to adopt public health guidelines speedily to deliver critical management or prevention strategies to clinicians to address rapidly spreading or evolving infectious diseases. Therefore, the objective of this project was to create a CDS solution that could be easily integrated using interoperability standards with widely‐used EHR systems in the United States. An interoperable solution comprising an external CDS service that is hosted centrally but integrated into clinical workflows via the EHR system presents potential benefits. For example, external CDS services can be adopted at scale as the development and testing of CDS logic do not have to be performed at each healthcare organization. Similarly, the updates to clinical practice guidelines can be disseminated rapidly by updating the CDS service centrally. There are potential challenges and disadvantages of external CDS services, such as slower performance from network delays, data privacy and security concerns, availability and variability in the encoding of data in the EHR, and the need to adapt the service to local workflows and varying requirements across healthcare organizations. There may also be concerns about trusting recommendations from an external CDS service that is not developed or governed by the healthcare organization [8].

An early study on external CDS services demonstrated its feasibility [9]. However, the authors also identified significant challenges related to governance, semantic interoperability, and usability. The HL7 CDS Hooks standard was subsequently developed to address the interoperability issue [10]. Solutions with CDS Hooks, integrated with the EHR, were successfully used to increase medical calculator use at the University of Utah [11], provide drug‐allergy checking in South Korea [12], increase HIV screening [13], and identify patients for genetic evaluation of familial cancer [14]. Other prototypes have been developed and used in test environments for drug–drug interaction checking [15] or genomic testing [16, 17], but have not been used clinically. Nevertheless, little is understood about the issues related to governance or usability of a CDS service that is utilized by multiple organizations. In this study, we also sought to further understand such issues by collaboratively designing a CDS solution, implementing the solution at two healthcare organizations, publishing the CDS knowledge artifacts as open‐source, and using interoperability standards.

## 2 Methods

The project team comprised PHII, CDC, Elimu Informatics, Yale New Haven Health (YNHH), Mass General Brigham (MGB), and Northwestern University. The latter three organizations provided clinical and EHR interoperability expertise. YNHH and MGB were the sites where the pilot of the CDS solution was conducted in 2024. Elimu Informatics provided informatics expertise and was responsible for the technical development and hosting of the CDS solution. CDC and PHII provided funding, expert knowledge on public health, and the disease‐specific guidelines.

### 2.1 Interoperability Approach

After consideration of the clinical problem and the potential CDS implementation options available in current EHRs, we chose to implement the CDS as alerts to clinicians in emergency departments or urgent care clinics when treatment for gonorrhea was missing or inappropriate, or when either screening for HIV or sharing information on PrEP was needed in patients with GC.

We decided to use the CDS Hooks standard to implement a scalable solution that interoperates with EHRs, as it enables the creation of the CDS solution and maintenance of the knowledge centrally in a cloud‐hosted service. At the time of our study, adoption of the CDS Hooks standard was limited to a small number of EHR products, which had implemented only a subset of its features. While the EHR at our study sites provided CDS Hooks capability, its limited implementation impacted the design of the CDS solution.

### 2.2 Knowledge Translation Approach

We followed the multilayered framework [18] for translating the STI, HIV testing, and PrEP clinical guidelines [4, 5] into computable knowledge artifacts required for the CDS solution. The framework supports stepwise translation of the knowledge from narrative clinical guidelines to semistructured recommendations, then to structured recommendations, and finally results in executable CDS knowledge.

#### 2.2.1 Creating Semistructured Recommendations

The scope and design of the CDS was the result of collaboration in our multidisciplinary team during the creation of the semistructured recommendations. The public health experts and clinicians identified the initial scope of CDS. Clinicians from each site participated in meetings to review the semistructured recommendations and to provide insight on workflows, data availability, feasibility, and clinician acceptance of the CDS intervention.

**FIGURE 1:**
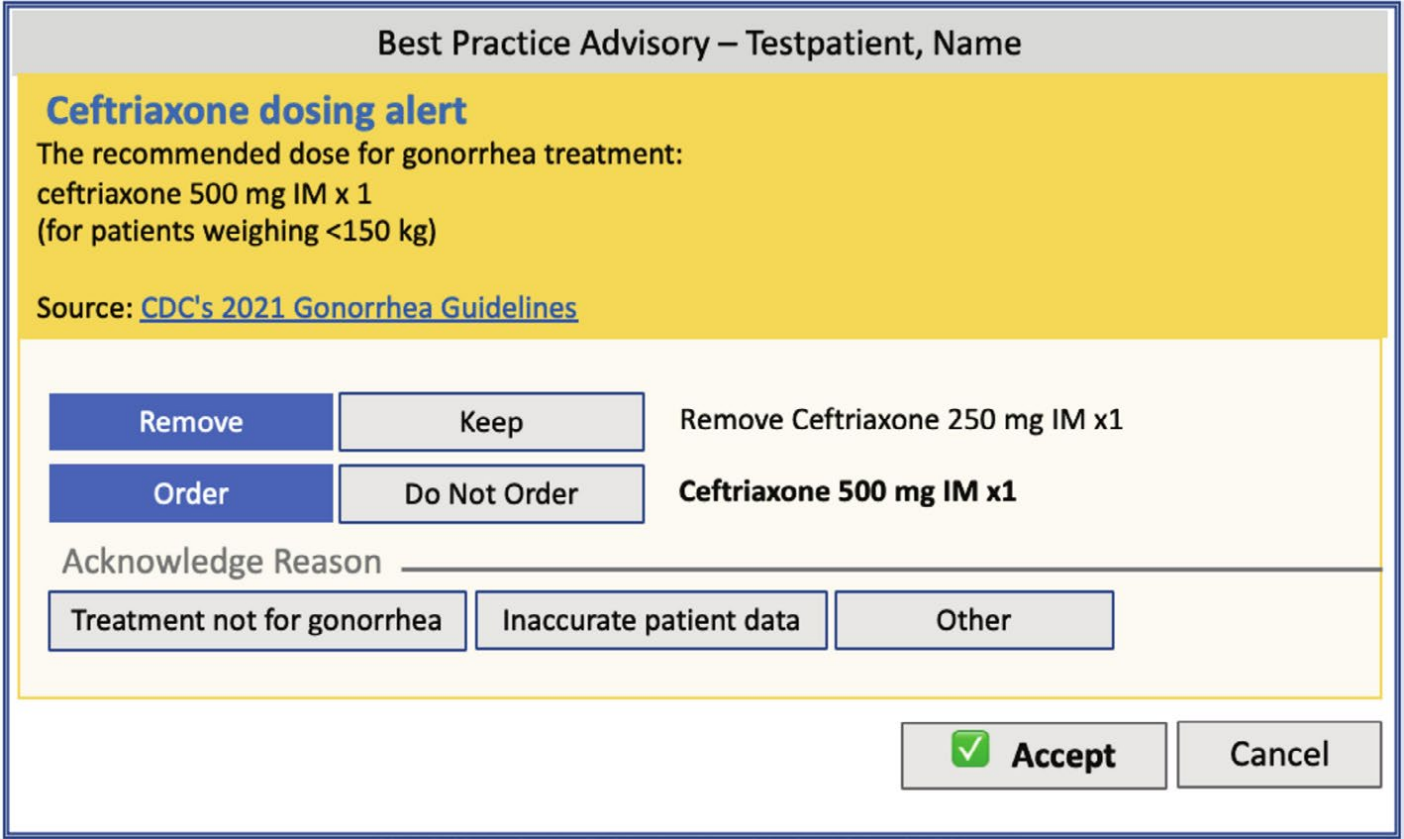
A mockup of an alert dialog to be displayed to the clinician ordering ceftriaxone with a dose inconsistent with the STI treatment guidelines.

**FIGURE 2:**
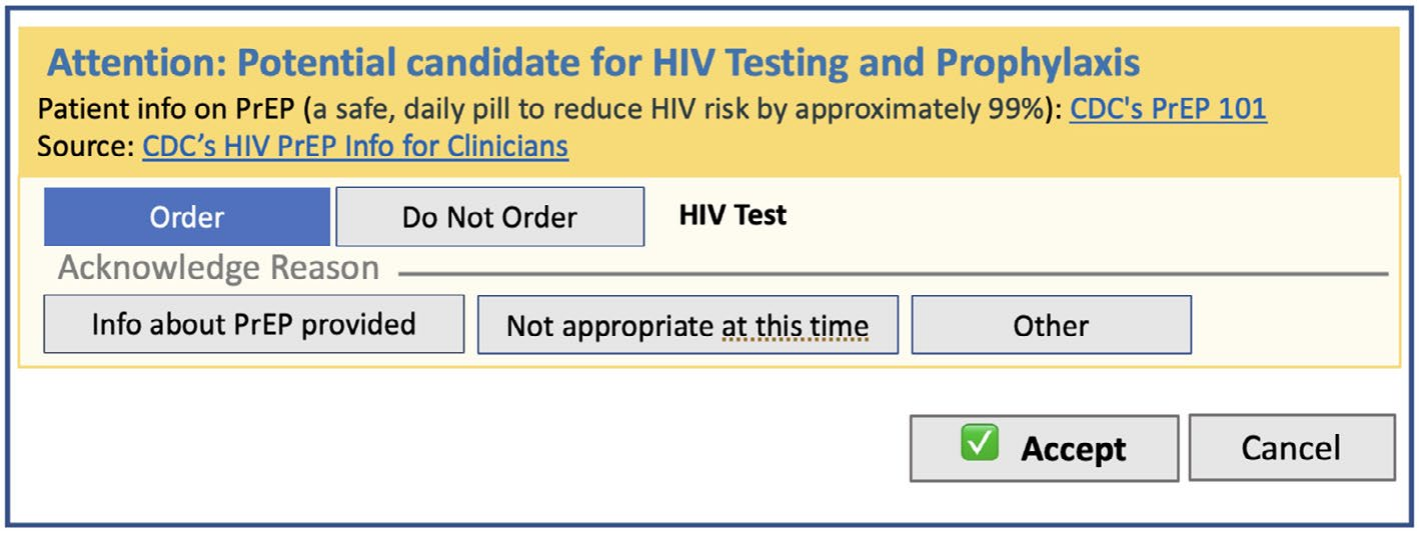
A mockup of an alert displaying HIV testing and PrEP recommendations; text describing PrEP is borrowed from another study on PrEP CDS and predates availability of injectable forms of PrEP [19].

We created mockups of the CDS to obtain feedback from the clinicians and the public health experts (Figures 1 and 2). The mockups were designed to be implementable in the EHR systems of the partnering clinical sites. The alerts provide (a) textual guideline recommendations for treatment, (b) preselected options for the CDS to remove an existing antibiotic order and draft an order with a guideline‐recommended antibiotic and dose, and (c) a list of potential reasons for the user to explain the override of the recommendations. Feedback from the stakeholders on the mockups was used to refine the CDS scope and logic.

The team eventually selected CDS for ceftriaxone dosing because it was one of the key updates in the STI treatment guidelines. The semistructured recommendations were drafted for the following scenarios, describing clinical settings, patient data needed, events that trigger the CDS, recommended actions, and explanations:

1. *Improper Treatment of Presumptive GC*: CDS is triggered by the presumptive treatment of gonorrhea for a dose that was either too high or too low based on the patient's body weight. Following clinician recommendations, presumptive treatment was inferred by the signing of an order for intramuscular ceftriaxone in the absence of laboratory confirmation. The CDS suggested guideline‐recommended antibiotics and doses customized to the patient for body weight, possible pregnancy, and drug allergies.
2. *Inadequate Treatment of Confirmed GC:* CDS is triggered by inadequate treatment of laboratory‐confirmed gonorrhea infection in the previous 6 weeks. Medication orders of all possible acceptable regimens of gonorrhea treatment (weight‐based ceftriaxone, cefixime, and a combination of gentamicin and azithromycin) are considered in assessing the treatment. The CDS suggestions were similar to those in the previous scenario.
3. *HIV Testing and PrEP Information for GC:* For patients who met criteria for the two scenarios above, additional CDS displayed: (a) a suggestion for an HIV antigen/antibody test, for patients who had not had one in the past 2 weeks, and (b) a link to provide the patient with information on PrEP, if desired. Patients with a recorded diagnosis of HIV in the problem list of their EHR or reported recent use of PrEP medications were excluded from these recommendations.

CDS for testing and treatment of chlamydia coinfection was excluded due to concerns about the complexity of the logic and the usability of the resulting CDS. Chlamydia has many treatment options, and implementing logic for those management recommendations, including checking for contraindications, was not feasible in the scope of our project. Providing informational text only was deemed an undue increase in the provider burden in an emergency department setting.

Accurate detection of pregnancy by available structured data was decided to not be feasible after discussion with clinicians, as available laboratory result data and diagnoses are highly variable and would have to rely on heuristics to determine current pregnancy status [20]. Instead, the CDS displayed additional text recommendations for potential pregnancy in women of childbearing age. Effort is ongoing to provide pregnancy status data via an HL7 Fast Healthcare Interoperability Resources (FHIR) Application Programming Interface (API) [21]. When this API can reliably provide current pregnancy status, the CQL logic and advice can be modified to be more specific for pregnant women. We considered including referral to an infectious disease specialist as an order option for some complex scenarios. However, we did not include that option, as referrals in the emergency department are typically ordered during discharge and not during medication order entry.

#### 2.2.2 Creating Structured Recommendations

**FIGURE 3:**
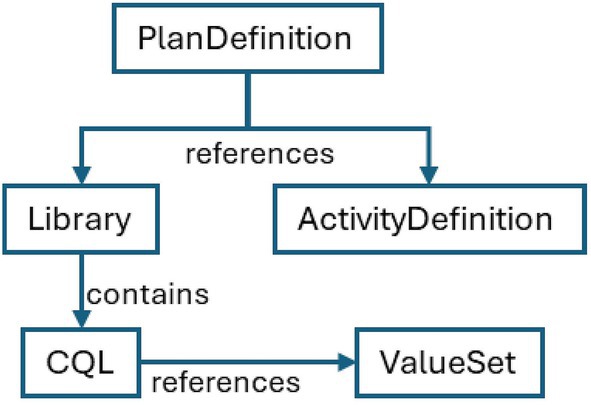
The FHIR artifact types to define the knowledge in the Structured Recommendation layer.

To align with interoperability standards, we created structured recommendations based on the HL7 FHIR Release 4 standard. Specifically, we followed the Clinical Reasoning module [22] to translate the semistructured recommendations to the following FHIR resource types: PlanDefinition, Library (Figure 3), ActivityDefinition, and ValueSet. Briefly, a PlanDefinition models an overall CDS scenario. The PlanDefinition references one or more Library resources, which contain the data retrieval expressions for data to be used in the CDS, and the logic expressions to determine if the CDS applies to the patient and to customize the recommendations. Expressions in the Library are written in HL7's Clinical Quality Language (CQL) [23]. The PlanDefinitions also reference ActivityDefinitions, which model the actionable recommendations. ValueSets are collections of codes, typically from standard terminology systems, that are used with the expressions in the Library to identify specific data elements needed by the CDS logic.

Two PlanDefinitions were created for this project: one for the presumptive treatment scenario and one for laboratory‐confirmed gonorrhea. The logic and actions for the HIV testing and PrEP alert were incorporated in the PlanDefinitions as these recommendations were only displayed in conjunction with those for gonorrhea treatment.

ActivityDefinitions were created for each possible suggested order, which were exclusively medication orders (a FHIR MedicationRequest resource) for the gonorrhea treatment domain and a lab order (a FHIR ServiceRequest resource) for the HIV testing and PrEP domain. The ActivityDefinitions included codes using standard terminologies such as RxNorm for medications and SNOMED‐CT for diagnostic test orders.

Multiple value sets of standard codes were created for the data retrieval expressions, for patient history of medications, allergies, and test results. We first checked the National Library of Medicine's Value Set Authority Center (VSAC) [24] for the existence of published value sets that could be reused in this project. While there was some overlap between our needs and existing value sets, most value sets were found to be too broad in their content and therefore unsuitable for a narrowly‐focused project (e.g., listing all STI tests rather than just GC tests). Ultimately only one published value set of HIV diagnosis codes was able to be reused. Other value sets from VSAC could not be reused due to the need for specificity in medication routes, body sites, and laboratory tests for this project. Medication value sets were created by a proprietary tool developed by our team, as we found this tool to be more efficient to search for and add medication codes by different categories. All other value sets were created directly within VSAC. Value sets containing SNOMED codes were created as intensional (rule‐based) ones using the hierarchy of parent–child relationships. Value sets containing LOINC codes were created as extensional (enumerated lists), with search and selection of individual codes. Value sets can be obtained as FHIR resources from VSAC and from our source code repository.

#### 2.2.3 Implementing the Executable Recommendations

Our CDS software implemented the Clinical Reasoning module specification, which describes how FHIR knowledge artifacts such as PlanDefinition can be used to implement CDS Hooks services [22]. Since we followed the same specification when creating the structured recommendations, we were able to use the latter as our executable recommendations.

However, a few modifications were necessary to the knowledge artifacts created in the structured recommendation layer. For example, EHR proprietary terminology codes were added to the ActivityDefinitions to map the recommended actions to orderable medications and tests in the EHR, thereby enabling the user to follow the CDS advice by simply accepting the order in the CDS prompt screen. Another change made was in the logic expressions in CQL to correctly detect medications in medication orders. FHIR allows flexibility in how medication codes are embedded in a medication request resource. Our structured recommendation logic needed to be modified to accommodate the pattern used by the EHR for certain injectable medications.

#### 2.2.4 The CDS Service

We developed the CDS service using several open‐source components. The API for the CDS Hooks service was implemented using Kogito [25], a framework for creating business automation software. The overall functionality of the service was specified as tasks in a business process in the Business Process Modeling Notation (BPMN) standard [26]. The business process specified tasks such as parsing and validating the CDS request from the EHR, evaluating the patient data against the executable knowledge artifacts, filtering responses to allow custom CDS for some sites (e.g., one of the study sites did not display the alerts for HIV Preexposure Prophylaxis). This process is executed by the Kogito engine. The service provided two API endpoints, one each for presumptive treatment and treatment of lab‐confirmed gonorrhea. Both of these services also provided CDS for HIV screening and PrEP prescriptions.

A custom module was added to the CDS engine to evaluate the logic specified in the FHIR knowledge artifacts and create “cards,” the format in which the CDS response is sent from the service to the EHR. This module utilized the open‐source CQL execution engine [27] from the Clinical Quality Framework project.

The CDS service obtained the knowledge artifacts from a FHIR server we refer to as the Knowledge Repository. The Knowledge Repository used the open‐source HAPI‐FHIR server [28]. In addition to the knowledge artifacts described above, the Knowledge Repository server also stored standard terminology systems to support evaluation of value sets. The standard terminologies were loaded as CodeSystem resources and included LOINC, RxNorm, SNOMED‐CT, and ICD‐10‐CM.

**FIGURE 4:**
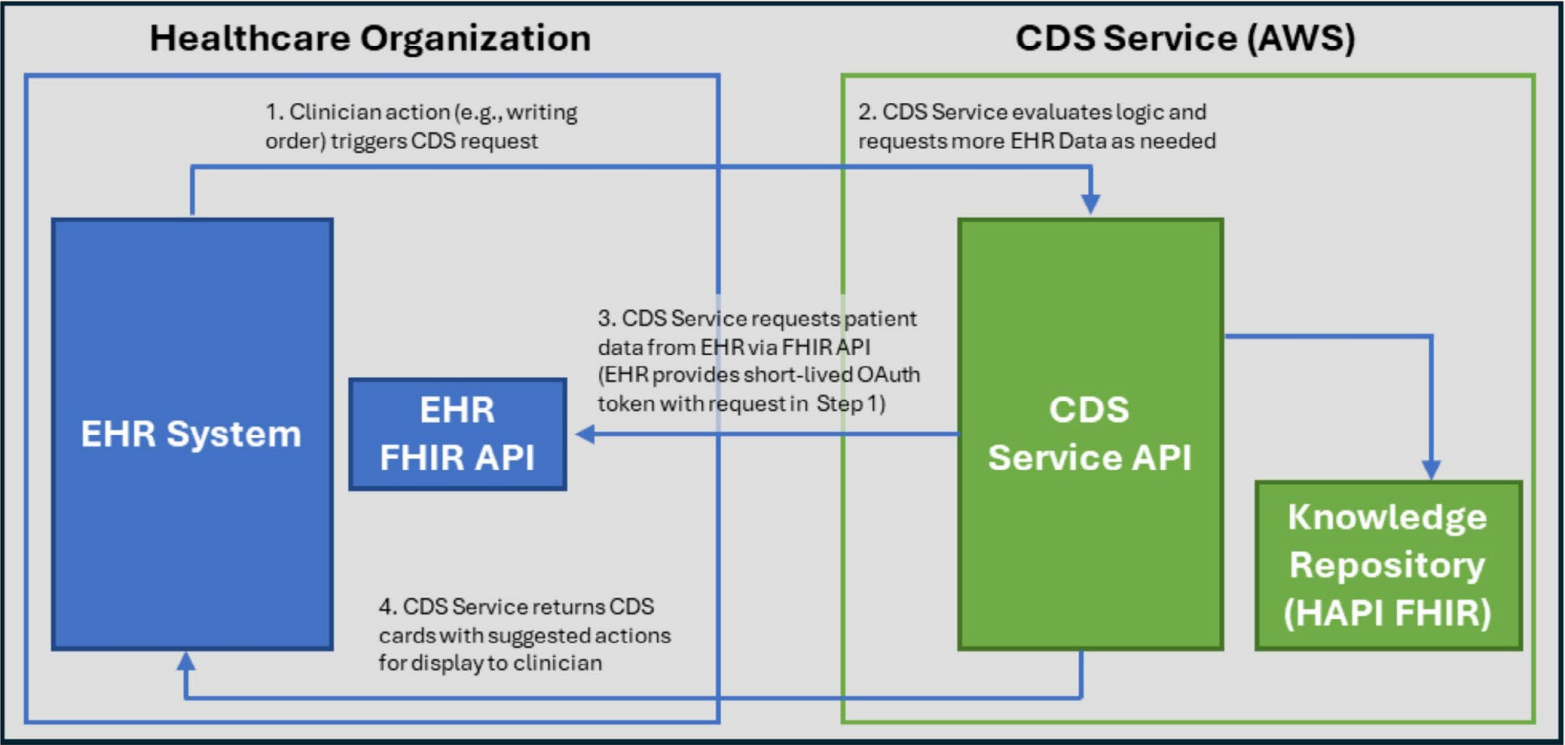
The flow of a CDS request between the EHR and the CDS service.

Figure 4 explains the flow of the service. A specific action in the EHR system, such as a clinician signing an order for ceftriaxone to be given intramuscularly, triggers a request to the CDS service's API. The request includes contextual data such as the draft order and the FHIR Patient resource. The service validates the request and then hands it off to the CQL engine to evaluate the patient data using the knowledge artifacts. The CQL engine obtains the artifacts from the knowledge repository. If additional data are needed (e.g., body weight), the service obtains it from the EHR's FHIR API using the server address and authorization token provided by the EHR with the CDS request. The CQL engine evaluates membership of codes in a value set using APIs provided by the knowledge repository. If cards are created by the CQL engine, those are returned to the EHR by the CDS service's API. The EHR then displays those cards to the clinician.

The CDS service was deployed in the cloud in Amazon Web Services (AWS). The system was configured to meet the regulatory requirements for security and privacy of protected health information (PHI). One of the key implementation choices was to not record any PHI in the cloud‐hosted CDS service. In other words, the CDS service obtained patient data from the EHR's FHIR API, used it to evaluate the CDS logic, and then discarded the data it had obtained after sending the CDS card back to the EHR.

#### 2.2.5 Testing and Piloting the CDS

The CDS was tested at multiple steps, with each step having different constraints and goals, as described in Table 1.

**TABLE 1:** Tests of the CDS were conducted at multiple steps with different objectives.

Testing steps	Purposes	Constraints and limitations
Logic testing	To evaluate if logic is syntactically correct and programmed as specified	Only evaluates the logical relationship of concepts as encoded in CQL, without testing any data transmission.
Developer sandbox testing	To perform full integration testing of the CDS service and knowledge artifacts. This step can be automated.	Tests integration with data formats and query capabilities that may not match those of the EHR FHIR API. Therefore, performance may not be equivalent to subsequent steps.
EHR acceptance testing	To assess integration with EHR, performance, and usability	Requires manual data creation or updates often to satisfy timing criteria in CDS logic. Cannot be automated.
Production testing	To ensure CDS is working as in the previous step of testing, but with real patient data.	Has limited testing capability, as artificial cases cannot be created, by policy, in the production EHR.

The CQL logic was developed and tested using an open‐source CQL plugin module [29] for Visual Studio Code (Microsoft Corporation, Redmond, WA). The plugin module allows the developer to test expressions in a CQL file using FHIR resources stored in the file system. The FHIR resources contain patient data and value sets. The results are manually inspected by the developer, who can then make necessary changes to the CQL expressions.

The second level of testing was an integrated test in a sandbox of the complete cloud‐hosted CDS service. For these tests, we used a public FHIR server as a proxy for the EHR's FHIR API, as well as testing in our own FHIR server. We created a set of test patients in the EHR FHIR server. The CDS Hooks requests were sent through Postman (Postman Inc., San Francisco, CA), a graphical tool for interacting with APIs. The API responses, which now included CDS Hooks cards, were used to identify any issues with the knowledge artifacts and the CDS service software. While these tests can be automated to run every time a new version is deployed, for this project, we limited them to manual testing.

Next, we conducted integration testing in the EHRs at both of our clinical sites. Prior to the integration testing, the information systems departments at both sites conducted a security review of the software, following their respective protocols for integrating third‐party software with their EHR systems. Detailed testing of CDS was conducted at YNHH, while the testing at MGB was focused on demonstrating the portability of the CDS Hooks solution. Once the CDS service was integrated with YNHH's EHR system in its test environment, meetings were held twice weekly for testing. Sample patients were created in the EHR, and then an analyst with a nursing background simulated the target actions of clinicians. Team members collaboratively reviewed the output. Relevant system logs were inspected for errors. Several rounds of testing were conducted. Issues in various components of the overall system were detected and addressed. These issues were related to network security, coding of data in the EHR, CDS performance, and aspects of the CDS Hooks standard implementation in the EHR. The test plan comprised 31 cases covering common variations we expected to see. For example, for the presumptive treatment scenario, patient scenarios were created with and without beta lactam allergies, weight less than and greater than 150 kg, and with relevant age and sex characteristics. For the laboratory‐confirmed gonorrhea scenario, the plan contained patients with the same characteristics as well as variations on recent gonorrhea test results and medication histories (e.g., a patient with a positive urogenital gonorrhea test and a record of an order for ceftriaxone 250 or 500 mg). The plan was created such that every specific component of the logic was tested at least once, including scenarios that tested if appropriate exclusions were being applied (e.g., including a test patient with age less than 13 years to ensure that the CDS card was not created). Testing was conducted iteratively until all issues were resolved.

While we could not conduct formal performance testing with a large number of test cases due to technical reasons, we performed ad hoc tests and optimized the CDS engine (by caching compiled CDS knowledge artifacts in memory on server start up) and the CQL logic to achieve a response time of under 3 s. The rate limiting step was in the CDS service retrieving patient data from the EHR. We modified the CDS engine to retrieve data in parallel and optimized the CQL expressions to limit the volume of data requested.

Testing was also conducted in MGB's EHR test environment with a small set of cases. The purpose of these tests was to demonstrate that the same service was able to be successfully executed from both environments.

After extensive verification in this test environment, the system was implemented in the production EHR system at YNHH. At first, the CDS was activated in “silent” mode, in which the service was called, and cards were created and returned from the service to the client, but were not presented to the clinicians in the EHR. This period of behind‐the‐scenes implementation was an additional level of testing to examine when cards were created with real patient data to assess if the system was generating cards appropriately, and no errors were created in the service. Additional revisions were made in the client's configuration based on this testing.

After testing in silent mode was performed for several weeks, the CDS went live, and the cards were presented to the 19 clinicians working in the YNHH emergency department during the duration of the pilot. The pilot period was of 3 months duration and conducted with approval of the Institutional Review Board at YNHH. This activity was reviewed by CDC, deemed not research, and was conducted consistent with applicable federal law and CDC policy. At the conclusion of this planned pilot period, the service was turned off. During the pilot period, the CDS was triggered a total of 522 times. Of these, the presumptive gonorrhea treatment CDS was triggered 250 times and returned CDS cards 19 times. The laboratory‐confirmed gonorrhea CDS was triggered 272 times and returned cards 14 times. Cards providing recommendations related to HIV screening and considerations for PrEP prescription were returned a total of 33 times.

The CDS software and knowledge artifacts are available as open‐source from the Github source code repository [30]. The value sets have been published to VSAC. A video demonstrating the service is also available [31]. It should be noted that the service deployed for the study had some differences from the open‐source software.

1. The service implemented Json Web Token authentication as suggested by the CDS Hooks specification to authenticate the EHR system that was invoking the CDS service. This feature is not enabled in the open‐source code as it uses proprietary source code. Authentication can be implemented by those desiring this capability.
2. The service tracked usage for purposes of the study. The tracked data included details of requests and responses. The usage was anonymized and PHI was not stored with usage logs.

## 3 Discussion and Conclusions

A multi‐disciplinary project team successfully developed an approach for implementing STI treatment and HIV prevention guidelines as CDS. Table 2 summarizes the strategies we used that could guide the future development of CDS based on public health guidelines.

**TABLE 2:** The strategies we used to implement CDS based on public health guidelines.

CDS scope	The scope of the CDS solution was based on public health importance of a recommendation, feasibility of implementation using contemporary technologies, and priorities of clinicians and patients.
Team	A multidisciplinary team was composed with expertise in public health, infectious disease, clinical informatics, knowledge representation, interoperability standards, and EHR implementation.
Translation approach	A progressive and iterative translation of guidelines to executable CDS allowed key decisions to be made at appropriate stages and balance public health priorities, technical implementability, and provider burden.
Transparency	CDS knowledge artifacts were specified using knowledge representation standards and published as open‐source to ensure transparency of the CDS logic.
Dissemination	Interoperability standards were used to integrate the CDS with EHRs to facilitate easier and faster implementation of the CDS at healthcare organizations.
Testing	The CDS was tested progressively in different environments. This allowed comprehensive testing while addressing challenges and limitations in each environment.

We used a collaborative and iterative approach with a multidisciplinary team in this project. The multilayer knowledge translation framework organized our activities with scoping and design by the public health experts and clinicians during the semistructured recommendation creation, the encoding of CDS knowledge by informaticians during the structured knowledge creation, and integration and clinical introduction by the informaticians, implementation analysts, and clinical champions during the executable CDS implementation.

We chose to encode the CDS knowledge using interoperability standards, specifically FHIR and CQL to promote transparency, and published the CDS knowledge as open‐source. This transparency in the logic is essential for a CDS service that implements public health guidelines to be accepted and adopted widely by healthcare organizations and their clinicians.

Our results suggest an interoperable external CDS service may be an option for deploying or updating CDS widely or rapidly. Using the CDS Hooks standard, our cloud‐based CDS service could obtain patient data from the EHR to execute the CDS logic. The CDS service could also deliver actionable recommendations, that is, clinicians could accept or refine appropriate medication and diagnostic testing orders in their EHR from the CDS card that was generated in the CDS service. However, additional progress on the CDS Hooks standard, its implementation, and adoption are needed to realize this potential. This might include development of new hooks and making existing hooks more configurable, as well as wider adoption of CDS Hooks and their full range of features by EHR vendors.

Extensive testing is key to ensuring the CDS logic performs effectively and integrates with EHR systems appropriately. The use of an external CDS service required us to adopt a tiered progressive testing approach due to varying requirements and constraints in each environment. The tiered testing approach also provides an efficient approach when external CDS must integrate with EHRs at multiple sites.

This study had important limitations which may affect the generalizability of our findings. While we integrated our service with two EHRs, both organizations' EHRs were from the same vendor. This is because at the time of our study, very few EHR products supported CDS Hooks. If other EHR products support a different subset of CDS Hooks features than tested in the pilot, our CDS service may not function in that EHR without modifications to the knowledge artifacts. Furthermore, we were able to conduct clinical testing in a live environment at only one organization. Testing at other organizations may have revealed additional issues with implementation. We also tested only the CDS modality, that is, alerting a user with a prompt on the screen. There may be other modalities for CDS that are supported by interoperability standards such as SMART on FHIR. Those modalities may be better suited for other use cases. We did not study governance issues in developing the CDS service, updating the CDS with revised content or adoption of the initial or updated version of the CDS at a healthcare organization. These are important issues that must be addressed in a nonresearch setting.

We explored the use of CDS interoperability standards to integrate an external, centralized CDS service with EHR systems. This approach shows promise as a means to rapidly deploy and update CDS based on public health guidelines. However, more work is needed to scale such guideline implementation efforts. Wider adoption of the standards in EHR systems is a prerequisite. Scaling will also require infrastructure for knowledge translation and trusted organizations to deliver reliable CDS services. Additional studies are needed to further understand the functional requirements, create governance frameworks, refine the interoperability standards, and develop scalable processes and technologies to implement CDS for public health guidelines.

## Funding

The publication and project described were supported by Cooperative Agreement Number NU38OT000316 from the Centers for Disease Control and Prevention. Its contents are solely the responsibility of the authors and do not necessarily represent the official views of the Centers for Disease Control and Prevention.

## Conflicts of Interest

Aziz Boxwala owns stock in Elimu Informatics. The remaining authors have no relevant conflicts of interest to disclose.

## Supporting information


**Video S1:** STI CDS system: Demonstration of API.

## Data Availability

The data that support the findings of this study are available from CDC and clinical partner sites. Restrictions apply to the availability of these data, which were used under license for this study. Data are available from the author(s) with the permission of CDC and clinical partner sites.
